# First Report of* Echinococcus ortleppi* in Human Cases of Cystic Echinococcosis in Poland

**DOI:** 10.1155/2019/2474839

**Published:** 2019-04-08

**Authors:** Monika Dybicz, Piotr K. Borkowski, Maurycy Jonas, Dariusz Wasiak, Piotr Małkowski

**Affiliations:** ^1^Chair and Department of General Biology and Parasitology, Medical University of Warsaw, 5 Chałubińskiego Street, 02-004 Warsaw, Poland; ^2^Department of Infectious Diseases, Tropical, and Hepatology, Medical University of Warsaw, 37 Wolska Street, 01-201 Warsaw, Poland; ^3^Department of General and Transplantation Surgery, Medical University of Warsaw, 59 Nowogrodzka Street, 02-006 Warsaw, Poland; ^4^Department of Surgical and Transplantation Nursing, Medical University of Warsaw, 59 Nowogrodzka Street, 02-006 Warsaw, Poland

## Abstract

Cystic echinococcosis is considered as an emerging zoonosis that can develop asymptomatically for years, clinically nonpathognomic. The disease is of public health importance due to often late, difficult diagnostics, uncertain results of treatment, the need to remove hydatid cysts surgically in advanced cases, and poor prognosis in untreated patients. Six Polish female patients with diagnosed cystic echinococcosis (CE) were examined. DNA extracted from the liver and lung samples served for amplification of mitochondrial* nad1* gene fragment. Sequence alignments of 5 isolates showed identity with the pig strain,* Echinococcus canadensis *G7. One case was in 100% identical with* Echinococcus ortleppi* G5, the cattle strain. These data demonstrate first report of* E. ortleppi*, regarded as extinct species, causing human cystic echinococcosis in Poland, where the most frequent causative agent of human CE is* E. canadensis*.

## 1. Introduction

Cystic echinococcosis (CE) is a globally distributed zoonotic disease caused by* Echinococcus granulosus *sensu lato which is a member of the Taeniidae family. In the life cycle of* Echinococcus *species, which is mostly domestic, dogs are the definitive hosts; however, wild canids (dingoes, wolves, jackals, coyotes, and red foxes), felids, and hyenids can also become definitive hosts in the transmission cycle. The adult worm lives in the small intestine and delivers eggs excreted with the stool. When intermediate hosts (sheep, goat, swine, cattle, or accidentally human) ingest eggs, they develop into hydatid cysts in the internal organs. The cysts in humans develop most commonly in the liver, and then in lungs, brain, heart, and bones. In humans, the disease usually develops asymptomatically for years until growing cysts start to press on the surrounding tissues and give nonspecific symptoms. Treatment of CE is considered difficult, because of the involvement of the vital organs and possibility of dissemination of fluid with protoscoleces during the surgery. Both deficient resection and bladder rupture may contribute to anaphylaxis, recurrence, and multiple secondary CE [1–3].

Echinococcosis is regarded as an emerging disease occurring worldwide with the highest prevalence in parts of Eurasia, North and East Africa, Australia, and South America [4–8]. Molecular analysis, based mostly on mitochondrial genes [9–13], have demonstrated that* E. granulosus* is a complex of many species differing in host specificity, pathology, development rate, and sensitivity to chemotherapeutics. The taxonomic revisions have led to revealing* E. granulosus* sensu stricto (G1-G3 genotypes),* Echinococcus equinus* (G4),* Echinococcus ortleppi* (G5),* Echinococcus canadensis* (G6-G10), and* Echinococcus felidis* (“lion strain”) [11, 14].

*E. ortleppi* was originally described as* E. granulosus* in dogs from South Africa [15]. The essential differences of* E. ortleppi* in development and morphology, compared to other species, have resulted in the later description of the distinct genotype as a separate species, related most closely to* E. canadensis*.* E. ortleppi* is considered as the cattle strain (G5), because it predominantly develops in cattle as intermediate host [16]; however, it has also been recorded in other animals such as sheep, pigs, goats, camels, buffaloes, deer, monkeys, and porcupine [17–23]. The cases of* E. ortleppi* causing human CE have been reported in South America, South Africa, Asia, and Europe, where animal and human [24–29]* E. ortleppi* infections have been documented rarely.

It is the first report on* E. ortleppi *cases, causing CE in Polish patients in Poland, who underwent surgery to remove hydatid cysts.

## 2. Materials and Methods

Fragments of cysts were collected from 6 female patients operated on in years 2016-2017 in the Department of Infectious Diseases, Tropical, and Hepatology, Department of Surgical and Transplantation Nursing, and Department of General and Transplantation Surgery, Medical University of Warsaw, Poland. The study was approved by the Bioethical Committee of Medical University of Warsaw, Poland. Certain patients with cystic echinococcosis had been under medical supervision for many years and were treated with albendazole. The cysts were removed surgically because of emerging complications. The collected patient data included age, gender, and place of residence. All 6 patients were females aged from 30 to 56 years. The examined samples represented by a fragment of a cyst were stored frozen at −20°C or fixed in 70% ethanol prior to molecular analysis.

### 2.1. DNA Extraction and PCR

The samples of examined isolates and* E. canadensis *G7 positive control (isolates JX266793 and JX266824) were rinsed with phosphate-buffered saline (PBS) several times to remove any ethanol residues and centrifuged at 5000 ×g for 10min. Each pellet was dissolved in 100 *μ*L PBS and genomic DNA was extracted using a NucleoSpin kit (Macherey-Nagel, Düren, Germany) following the manufacturer's instructions. Mitochondrial region of NADH dehydrogenase 1 (*nad1*) gene was amplified by PCR using primers JB11 (5'-AGATTCGTAAGGGGCCTAATA-3') and JB12 (5'-ACCACTAACTAATTCACTTTC-3') [30]. The 50 *μ*L reactions comprised of 1*μ*L of DNA template, 50pM of each primer, 0.2mM of each dNTP, 1x PCR buffer containing 2.5mM MgCl2, and 1U of Taq DNA polymerase (Qiagen, Hilden, Germany). The following PCR was performed in a PTC-200 thermal cycler (MJ Research, Waltham, USA) in the following conditions: 3 min at 95°C followed by 35 cycles of 1 min at 95°C, 1 min at 50°C, 1 min at 72°C, and final extension at 72°C for 5 min [31]. The PCR products were separated by electrophoresis on a 2% agarose gel (MetaPhor, FMC BioProducts, Philadelphia, USA) and then stained with ethidium bromide and observed on a UV transilluminator. The* nad1 *gene fragments were purified and then directly sequenced in both directions using a BigDye Ready Reaction Cycle Sequencing kit and an ABI 3730 Genetic Analyzer (Applied Biosystems, Foster City, USA). Chromatograms were manually checked and edited using Chromas 2.0. The obtained sequences were aligned with others retrieved from NCBI GenBank using ClustalW2 (http://www.ebi.ac.uk/Tools/clustalw2).

## 3. Results

A total of 6 samples were obtained from female patients (at the age of 30-56 years) who underwent surgery to remove cysts located mostly in the liver, and a single cyst was found in the lung. The patients came from central Poland (4 patients) and the other 2 from villages of southeast and east Poland. The cysts ranged in size from 3 to 12 cm in diameter and were mainly sterile (type CE3 and CE4) (Table 1).

DNA extracted from all 6 samples and positive controls were used as the template in separate PCRs to amplify region of the mitochondrial NADH dehydrogenase 1. Each PCR produced a single band upon agarose gel electrophoresis. All isolates and controls were diagnosed as positive by amplification of* nad1 *fragment (~500 bp). The* nad1 *fragments were sequenced and compared with sequences of* Echinococcus *genotypes determined by using BLAST software (http://www.ncbi.nlm.nih.gov/blast). The sequences of 5 isolates showed 100% identity to* E. canadensis *G7, the pig strain. The sequence of 1 isolate was identical to the cattle strain* E. ortleppi *G5. All* nad1 *sequences were deposited in GenBank with accession numbers MH492787–MH492792.

## 4. Discussion

Human cystic echinococcosis is an emerging zoonotic disease occurring worldwide caused by the metacestode stage of* Echinococcus *species.* E. granulosus *sensu lato is a complex of many genotypes differing in pathology, development, sensitivity to anthelmintics, and intermediate host specificity, including sheep, goats, horses, cattle, pigs, camels, and members of the cervid family. Many distinct genotypes of* E. granulosus* s.l., marked as G1-G10, have been identified based on genetic diversity of the mitochondrial genes, mainly NADH dehydrogenase (*nad*) and cytochrome C oxidase (*cox*) genes [11, 14, 32]. The genotype description of* E. granulosus *G1 revealed that the sheep strain is the cosmopolitan genotype most commonly distributed in areas of extensive sheep farming and the predominant strain infecting humans; however, some other strains are also infective. In Poland, molecular studies proved that* E. canadensis* G7, the pig strain, is the main genotype infecting humans [31, 33, 34]. In many European countries, G7 genotype has often been identified due to the fact that pigs are raised on small farms in close contact with the breeders and definitive hosts. In this analysis, 6 samples of human liver cyst fragments were examined. All patients were of Polish nationality, living in central and east regions of the country. Genetic study of 5 isolates also confirmed G7 genotype, affirming that this strain has a significant role as the main aetiological agent of human cystic echinococcosis in Poland.

Interestingly, one isolate was identical to the cattle strain G5; this is the first report representing* E. ortleppi* case causing human CE of the liver carried out in Poland. This isolate involved a 38-year-old female who lives in a house with her own vegetable garden in close proximity to the forest allowing the forest animals to trespass. The patient is an office work with not a rich travel history; she visited Germany once. The female patient was diagnosed to have a cyst (12 cm in diameter) showing thick wall of about 3 mm in the right liver lobe. Using computed tomography scan, the cyst was described as probably decaying calcified and did not show any specific features being different from the cysts of* E. canadensis*, diagnosed most commonly in Poland. Infection with* E. ortleppi *causing human CE is rarely diagnosed, although G5 genotype has already been reported from humans in Netherlands, France, Argentina, Brazil, Mexico, India, Vietnam, and South Africa [24–29]. As it has been suggested by some authors,* E. ortleppi *may become extinct in Europe because of fewer opportunities for its transmission between cattle and dogs [16]. However, it has also been recorded in farm animals, ruminants, camels, buffaloes, deer, monkeys, and porcupine [17–23]; therefore, this Polish human CE case of* E. ortleppi* might have pig origin.

Molecular analysis of CE is not conducted frequently in Poland; therefore, it is impossible to determine if* E. ortleppi* is widespread in Poland or to evaluate the potential for human infection with this parasite. No studies were performed and the situation of genotype G5 in livestock in Poland is not known. Our identification of* E. ortleppi* infection in human enhances the need of systematic genotyping of* Echinococcus *cysts found in humans to determine the patterns of infection and pathology and expand the study of the strains being capable to infect human in Poland. In many areas of the country, pigs and cattle are bred on farms in close proximity to carnivorous definitive hosts, especially dogs, exposing humans to the eggs of the parasite. Genotypes* E. ortleppi* (G5) and* E. canadensis* (G7) are different in biology compared with some other strains of* Echinococcus*, for example, they have a shorter maturation period in dogs, which should be considered when planning the anthelmintic treatment regime for dogs. However, both strains can develop in humans, generating threats to the public health and the animal breeding.

## 5. Conclusions

In Poland, human CE is not frequent and mainly a result of infection with the pig strain of* E. canadensis* (G7). Our research represents the first record of human* E. ortleppi* infection, rarely detected in Europe. Furthermore, the future molecular studies of* E. granulosus* s.l. complex, inhabiting humans but also domestic and wildlife hosts, are attentive to provide important information on the population of* Echinococcus* genotypes and to better understand the aetiology and epidemiology of echinococcosis in Poland.

## Figures and Tables

**Table 1:** The description of cystic echinococcosis cases isolated from female patients in Poland. Abbreviations: USG – ultrasonography, CT – computed tomography scan, MRI – magnetic resonance imaging, ELISA – enzyme-linked immunosorbent assay.

Patient's age in years	City/district of patient	Location, number and type of cyst	Diagnosis data	Strain identification by sequencing
56	Warsaw (central Poland)	Liver single cyst (ø 7 cm), type CE3, treated with albendazole	USG	*E. canadensis*

38	Baranowo (central Poland)	Liver single cyst (ø 12 cm), type CE4, not treated pharmacologically	USG, Western blot positive for *E. granulosus*, ELISA negative	*E. ortleppi*

42	Niegowiec (central Poland)	Liver 2 cysts (ø 8 and 3 cm), type CE3, treated with albendazole	CT	*E. canadensis*

42	Zaolszynie (east Poland)	Liver 7 cysts (ø 8, 6, 4 (4 cysts) and 3 cm), type CE3 and CE4, treated with albendazole	MRI, USG	*E. canadensis*

30	Wrzelów (southeast Poland)	Liver 2 cysts (ø 5 and 4 cm), type CE4, lung single cyst (ø 4 cm), type CE3, treated with albendazole	CT, USG, serological test positive for *E. granulosus* and *E. multilocularis*	*E. canadensis*

38	Czarnowiec (central Poland)	Liver single cyst (ø 10 cm), type CE4, not treated pharmacologically	USG	*E. canadensis*

## Data Availability

(1) The sequences (accession numbers MH492787–MH492792) used to support the findings of this study have been deposited in the GenBank repository. (2) The methods data used to support the findings of this study are included within the article. (3) Previously reported primers data were used to support this study and are available at https://doi.org/10.1016/0020-7519(93)90065-7. These prior studies (and datasets) are cited at relevant places within the text as references no. [30].
